# Meat-inspection and Milk-inspection1Read at the seventeenth annual meeting of the Michigan Veterinary Medical Association.

**Published:** 1899-08

**Authors:** H. F. Palmer

**Affiliations:** Brooklyn, Mich.


### MEAT-INSPECTION AND MILK-INSPECTION.[1]

By Dr. H. F. Palmer,

BROOKLYN, MICH.

"Guard the food of the people and you guard their health."

We as a nation have just passed through a struggle that has cost the lives of many of her brave sons, and out of that struggle has grown a controversy in regard to the food of its army. Embalmed beef, rotten beef are the words upon the lips of many. We view these published statements, and record the word "awful," and at times are inclined to dethrone our own beloved Secretary of War. At the same time we allow a food to be given to our own family—to our own children—for which, for the number of deadly bacteria contained, the Cuban beef would be a sickly comparison.

We wonder at and abhor the death-rate of our wars, and think it is something terrible; but we rest content, and each year see more deaths from drinking tuberculous milk than if,

[1] Read at the seventeenth annual meeting of the Michigan Veterinary Medical Association.

to-day, England and our own country should be engaged in a deadly warfare.

No arguments are needed at this time and before such a gathering to show that inspection is necessary and will save many lives each year; but the main questions with which we are concerned are: Who shall inspect? What shall we inspect? How shall we inspect?

The people of the United States use more meat per capita than any other country save Australia, this being a section where meat is cheap and abundant Inasmuch as this constitutes one of the principal articles of diet it should be inspected. In 1881 our pork was prohibited entrance into Germany, France, and the principal European countries, because it was thought to be infested with trichinæ. In 1891 Congress directed the Secretary of Agriculture to inspect, previous to slaughter, all cattle, sheep, and swine, the carcasses of which were intended for interstate or foreign trade. To-day all the beef exported to Europe and the greater part of all pork-products and meat-products are inspected. To-day we send pure, wholesome meat to feed the Spaniards, while we are eating the uninspected, germ-laden products in order to cope with these same Spaniards.

The Bureau of Animal Industry is doing a grand work along the inspection line, but as yet a lack of sufficient means cuts short some contemplated work. This bureau has been in existence some fourteen years, and we can partially realize the scope of its work by glancing at its last yearly report.

Meat-inspection was in operation in one hundred and thirty-five abattoirs, located in thirty-five cities. Over fifty-one million animals were inspected, of which the inspectors rejected many thousand carcasses. They sent out enough tuberculin to test fifty thousand head of cattle, gave half a million doses of black-leg vaccine, reducing mortality in this disease from 15 to 1 per cent. They have given to the hog-raising section of our country a great boon in the serum-treatment of hog-cholera, whereby 80 per cent. of affected animals can be saved, and helped the cattle industry by working up a harmless dip for cattle, whereby the tick of Texas fever can be destroyed.

Of course, all this work necessitated the outlay of thousands of dollars; but here is one forcible illustration that a penny spent is one dollar saved. With an average cost of less than one cent a piece for inspection, who can value the saving to the people? Many lives were thus saved, to say nothing of the suffering and distress that was greatly decreased.

We demand that the meat sent to foreign countries shall be inspected; while we—yes, you and I—are compelled to eat the uninspected meat. There are many inconsiderate and unscrupulous butchers who stand ready to buy that which cannot be put on the foreign market, and prepare it for the home market; and they will do that thing just as long as there is no one appointed whose business it is to see that condemned meat is put in the tank with the offal and made into fertilizer.

The immediate charge of inspection is given to those veterinarians who have entered the service by a competitive examination. It has been proven that the persons obtained from such examination, one of the requirements of which is that they must be graduates of a regularly organized veterinary college, are more competent and efficient than non-professional men. The inspectors were placed in the classified service in 1894.

There are certain diseases among animals that render the flesh positively dangerous to use as food—such as anthrax, septic conditions, malignant œdema, and foot-and-mouth disease. Others may not be positively dangerous, but should be treated with suspicion, as tuberculosis, actinomycosis, Texas fever, swine-plague and any disease that causes a rise of temperature. Others, although not dangerous to use as food, would be considered very loathsome, as those that are drowned, smothered, and unborn, females in parturient state, and flesh containing parasites.

All animals should be inspected previous to slaughter, as many conditions are there found that would not be detected in the carcass. Fever, fatigue, exhaustion, starvation, and excitement can be readily detected on ante-mortem examination, and they all affect the quality of the meat.

Education is a great factor in inspectors' work. Those who never see the flesh prepared for food would be horrified to view certain parts that are perfectly wholesome.

A man may think there is no harm in the consumption of tuberculous milk or meat, as no case can be directly proven where such milk or meat caused the disease in the human. In this case they will not accept circumstantial evidence; but those same persons would be the twelfth man of a jury willing to incarcerate a man at Jackson for the rest of his natural life on circumstantial evidence alone, and that is no stronger in one case than another. Experiment has shown that pigs fed on tuberculous milk will contract the disease, and can we think the human family is less susceptible to such disease than our friend, the hog.

Milk is another important factor in the food-supply of our people. Some of us may be vegetarians and refrain from the use of meat, but all of us got our start in life by that one factor—milk, this being the only product of nature that combines all the elements requisite to a healthy condition. Milk is the natural food of all infants and invalids. Being consumed at that period of life when the body is so susceptible to disease, how careful ought we to be to see that our milk-supply is pure and wholesome.

Milk is the most universal product in use. It is estimated that it would take a tank twenty-five feet high and covering one acre to hold the supply used by our people in one single year.

Previously to the year 1870 milk was not known to carry the germs of disease, but it is now proven beyond a shadow of a doubt that milk is one of the disseminators of disease. The temperature just below the body temperature, the most natural one at which milk is kept, is the temperature best suited for the growth and multiplication of germs. Normal milk from a healthy cow is free from bacteria, but there are many ways in which infection may take place. The animal itself, the hands of the milker and the dust of the stable are each liable to share its quota of germs with the innocent milk that is being prepared for food. If milk could be obtained perfectly free from bacteria, its keeping properties and its high value as food would be assured; but when we think of millions of these germs in every gallon of commercial milk, any one of which may find a lodging place in our system and cause our death, should we not be a little careful of the kind of germ we are devouring?

The milk standard is set at the following figures by this State: 12.5 per cent. of total solids, 3 per cent. of butter-fat; specific gravity between 1029 and 1033; specific gravity of skimmed milk should be from 1032 to 1037, and may be sold from cans plainly labelled "skimmed milk."

However, at this time we are not as much concerned about the per cent. of butter-fat, whether we are buying the milk first drawn from the udder and letting the calf have the last and the best, and whether the milk-supply is drawn from a herd of pure-bred Jerseys or just grade cows; but we are the more concerned about the method of handling that milk, the healthiness of the animal producing it, and whether or no the man who milked that cow had any disease that could be transmitted to our family by means of that milk.

Tuberculosis, typhoid fever, cholera infantum, diphtheria, and scarlet fever are some of the dangers lurking in commercial milk. These dangers are perhaps greater in the use of milk than meat, for milk is commonly used in the raw state, while many of the germs of meat are killed by the cooking of that meat. It is said that cholera infantum, or milk diarrhœa, causes one-fourth the deaths of all infants, while one-fifth of the infants are victims of tuberculous milk. Is not this then a strong plea for universal inspection of milk?

It is an easy matter to sit down and outline ideal conditions for the handling of milk, but the commercial aspect hinders the carrying out of such an ideal. When we consider that the milk, coming, as it does, in sharp competition with uninspected milk, must be sold for a few cents a quart, we can realize why so much of our milk is uninspected. Educate the consumer, and he will be willing to pay the few added cents to each quart of milk in return for receiving pure, wholesome, and inspected milk.

And now a few words as to how inspection of both meat and milk could be bettered. Certainly the United States and each State should work in unison, so that there may be a strict uniformity of laws. All meat should receive the inspector's stamp before it comes to the consumer, even to that piece dealt out by the one-horse wagon that calls at your door. Perhaps this cannot be accomplished with the great number of slaughtering establishments as at present, but I would concentrate these establishments and have municipal abattoirs. There the small butcher who uses but one or two carcasses a week may have all the advantages of killing-floor, cooling-room, and inspector's stamp, as the dealer who uses his eight or ten a day. Inasmuch as inspection adds to the selling value of meat, the owner of these carcasses should pay the added cost of inspection.

All milk dealt out should also be inspected. This does not mean the milk alone, but the inspection of that milk should begin with the animal that produces it. She should be free from disease, in a thrifty condition, kept in clean, well-ventilated quarters, and fed on pure, wholesome food. Ensilage, decaying vegetable matter, and various other things are known to give a taint to milk. All things used about the milk and milk-room should be scrupulously clean. Attendants should be free from communicable diseases. If the milk, when first drawn, is Pasteurized or heated to 167° F., and kept there forty minutes by live steam, and then at once bottled, ready for the consumer, many of the deadly germs will be destroyed and no harm done to constituents of milk. Abandon the old form of milk-ticket, so that one day it will not be handled by a scarlet-fever patient and the next day by a person just in the right condition to help the growth of the few germs adhering to that same ticket.

Inasmuch as the health of our domestic animals plays so important a part in the health of our people, I would say, place a veterinary surgeon on every State board of health. The people need to be educated to know what veterinary science is doing and can do for them to better the sanitary condition. A virulent disease transmissible to man breaks out among our domestic animals. It is at once recognized by the competent veterinarian, measures are taken to prevent the spread of the disease, and who can tell the number of lives saved by this timely work. Education is the keynote in all this work. Educate the butcher, the consumer, the meat-dealer, and the dairyman, and you add one item to universal inspection. Give to the dairy schools of our country your hearty support, and the day will soon come when we can eat a piece of meat or drink a glass of milk and have no fear of consuming deadly germs.

